# Identification and analysis of deletion breakpoints in four Mohr-Tranebjærg syndrome (MTS) patients

**DOI:** 10.1038/s41598-022-18040-y

**Published:** 2022-09-02

**Authors:** Nanna Dahl Rendtorff, Helena Gásdal Karstensen, Marianne Lodahl, John Tolmie, Catherine McWilliam, Mads Bak, Niels Tommerup, Lusine Nazaryan-Petersen, Henricus Kunst, Melanie Wong, Shelagh Joss, Valerio Carelli, Lisbeth Tranebjærg

**Affiliations:** 1 Department of Clinical Genetics, Center of Diagnostics, Copenhagen University Hospital, Rigshospitalet, Copenhagen, Denmark; 2 Clinical Genetics Service, Laboratory Medicine Building, Southern General Hospital, Glasgow, Scotland; 3 Clinical Genetics, Human Genetics Unit, Ninewells Hospital, Dundee, Scotland; 4 Wilhelm Johannsen Center for Functional Genome Research, University of Copenhagen, Copenhagen, Denmark; 5 Department of Cellular and Molecular Medicine, University of Copenhagen, Copenhagen, Denmark; 6 Center for Genomic Medicine, Copenhagen University Hospital, Rigshospitalet, Copenhagen, Denmark; 7 Department of Otorhinolaryngology, Radboud Institute for Health Sciences, Radboud University Medical Center, Nijmegen, The Netherlands; 8 Department of Otorhinolaryngology, Maastricht University Medical Center, Maastricht, The Netherlands; 9 Department of Allergy and Immunology, The Children’s Hospital at Westmead, Sydney, Australia; 10 West of Scotland Centre for Genomic Medicine, Queen Elizabeth University Hospital, Glasgow, UK; 11 IRCCS Istituto delle Scienze Neurologiche di Bologna, Programma di Neurogenetica, Bologna, Italy; 12 Unit of Neurology, Department of Biomedical and NeuroMotor Sciences (DIBINEM), University of Bologna, Bologna, Italy; 13 Institute of Clinical Medicine, University of Copenhagen, Copenhagen, Denmark

**Keywords:** Molecular biology, Genetics, Clinical genetics

## Abstract

Mohr-Tranebjærg syndrome is an X-linked syndrome characterized by sensorineural hearing impairment in childhood, followed by progressive neurodegeneration leading to a broad phenotypic spectrum. Genetically MTS is caused by pathogenic variants in the *TIMM8A* gene, including gene deletions and larger contiguous gene deletions. Some of the latter involve the neighboring gene *BTK*, resulting in agammaglobulinemia. By next‐generation mate‐pair sequencing we have mapped the chromosomal deletion breakpoints of one MTS case and three XLA-MTS cases and used breakpoint-spanning PCR to fine map the breakpoints by Sanger sequencing. Two of the XLA-MTS cases presented with large deletions (63.5 and 27.2 kb), and the junctional regions were characterized by long stretches of microhomology, indicating that the events have emerged through homologous recombination. Conversely, the MTS case exhibited a small 2 bp region of microhomology, and the regions were not characterized by extensive microhomology. The third XLA-MTS case had a more complex breakpoint, including a 59 bp inverted insertion, thus at least four breakpoints were involved in this event. In conclusion, mate-pair library generation combined with next-generation sequencing is an efficient method for breakpoint identification, also in regions characterized by repetitive elements.

## Introduction

Mohr-Tranebjærg Syndrome (MTS; MIM: #304,700) is a rare X-linked recessive disorder characterized by progressive sensorineural hearing impairment in early childhood, followed by neurodegeneration leading to a broader phenotypic spectrum which may include ataxia, dystonia and visual disability^1,2^. Through linkage analysis MTS was mapped to Xq21.3-q22, and subsequently variation in *TIMM8A* (MIM: #300,356) was associated with the disease^2,3^. The *TIMM8A* gene is relatively small, consisting of two exons encoding a 97 amino acid protein, which functions as a translocase of the inner mitochondrial membrane^4^. A list of variants in *TIMM8A* have been described, including missense, nonsense, splice site variants and small insertions/deletions (indels) as well as partial and whole gene deletions^3,5–9^. Furthermore, contiguous gene deletions including *TIMM8A* account for a large proportion of the variants reported^5^. The contiguous gene deletions most commonly include the *BTK* gene (MIM: #300,300), which is located less than one kb downstream of *TIMM8A*, but larger deletions involving additional genes in the region have also been described^10,11^. Pathogenic variants in *BTK* cause immunodeficiency due to agammaglobulinemia (XLA). XLA is characterized by early onset of recurrent bacterial infections, profound hypogammaglobulinemia and markedly reduced circulating B cells. Clinically, contiguous gene deletions involving both *TIMM8A* and *BTK* include a phenotypic spectrum of MTS and XLA, known as XLA-MTS syndrome.

Technologies commonly used for studies of structural variants including larger deletions comprise methods such as microarrays, qPCR, and long-range PCR. Furthermore, in the past decade Next Generation Sequencing (NGS) sequencing-based technologies have emerged as powerful in detecting previously inaccessible variants. NGS-based strategies add certain advantages compared to microarrays in the detection of copy-number variants (CNVs), that includes deletions and insertions/duplications. Vergult et al. (2014) showed that mate pair sequencing can reliably detect the same copy-number variants as detected by array-comparative genomic hybridization (array-CGH)^12^. In addition, mate pair sequencing will uniquely reveal where and how a duplication is inserted, and it will reveal balanced rearrangements which cannot be detected by array-CGH. Long-range PCR combined with NGS is another efficient choice to analyze for CNV’s in candidate genomic regions especially in a small number of samples^13^. However, these strategies are limited to detect variants within the target sequence specified by the primers. Targeted panel and exome sequencing methods are useful for calling small germline variants and can in addition be used to detect CNV’s, but, as long-range PCR, have a low resolution for determining exact breakpoints of deletions outside the targeted regions. By contrast Whole Genome Sequencing (WGS), and long-read single molecule sequencing technologies such as Oxford Nanopore Technologies (ONT) are powerful NGS methods for also resolving deletions involving breakpoint in non-exonic regions including regions with repetitive regions and these methods are expected to continuous evolve^14,15^).

Deletions causing Mohr-Tranebjærg syndrome by including partial or whole gene deletion of *TIMM8A* have been published previous in about 20 patients/families, but only few describing the deletions down to the nucleotide level^5,9–11^. Arai et al. (2011) analyzed the genomic breakpoint in three XLA-MTS patients using methods such as array-CGH and long-range PCR. Here we describe one case clinically presenting with MTS and three cases presenting with XLA-MTS with contiguous gene deletion involving *TIMM8A* and *BTK*. The deletions were characterized using a combination of successive PCRs testing for the presence of amplified products from *TIMM8A* and surrounding genomic regions and fine mapped by mate pair NGS sequencing and breakpoint-spanning PCRs to sequence level.

## Results

### Identified breakpoints

**Figure 1:**
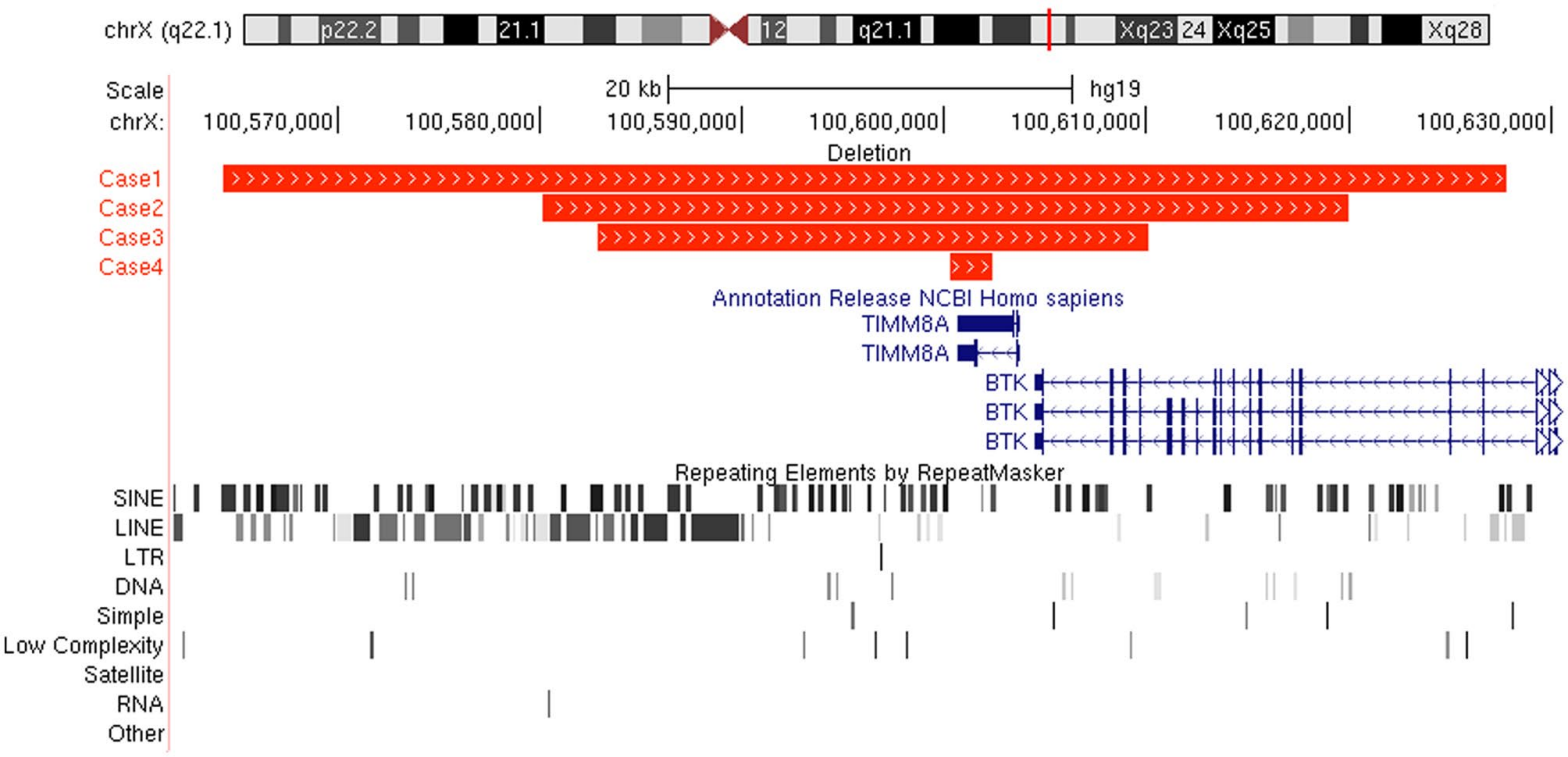
Overview of the *TIMM8A* and *BTK* gene region (hg19 chrX:100, 561, 900–100, 630, 700) with custom tracks showing the deleted regions of case 1–4, marked by red horizontal bars. The RefSeq gene track is shown in blue, and below repeating elements (long terminal repeat elements (LTRs), short and long interspersed elements (SINEs and LINEs, respectively)) are shown based on RepeatMasker information. For each of the four cases, minimum one of the junctional deletion regions were located within repeating elements.

In all four cases, deletion breakpoints were identified by a combination of successive PCR testing for the presence or absence of amplified products. Subsequently, mate-pair sequencing was carried out to allow precise placement of primers for successful break point-spanning PCRs (Fig. 1).

#### *Case 1* (XLA-MTS) 

Australian male patient, briefly mentioned in Tranebjærg, 2013, as patient 33 (18 months old) in Table 1^5^. Clinically, the boy presented with profound hearing impairment, and had cochlear implant (CI), as well as severe behavioral abnormalities and XLA. At age 16 he was diagnosed with autism spectrum disorder (level 3) attention deficit disorder, dysgraphia. His school performances were above competent level with minimal support in earlier periods, but with recent regression in his socio-emotional development to that comparable of a pre-school child. He had considerable separation anxiety and was regularly followed by a child psychiatrist and medicated for his psychiatric problems.

A de novo 63.5 kb deletion (ChrX: NC_000023.10:g.100564340_100627836del) spanning *TIMM8A* and exon 4–19 of *BTK* was identified. Initially, the deletion was identified using sequential PCR, amplifying genomic regions between *TAF7L* and *BTK* exon 1. Subsequently, mate pair sequencing and finally breakpoint spanning PCRs were used to delineate the deletion breakpoints. Both breakpoints were located in SINEs (short interspersed elements) Alu repeat regions. The mother of the proband did not harbor the deletion in DNA purified from blood, indicating a de novo origin.

#### *Case 2* (XLA-MTS)

Scottish male patient, born in 2000, briefly mentioned in Tranebjærg, 2013, as patient 34 in Table 1^5^. Clinically, the boy presented with deafness and some behavioral abnormalities, as well as XLA. No visual abnormalities were described at the age of 9 years. At age 21 he had chronic lung disease and bronchiectasis and mild intellectual disability with some behavioral issues including aggressive outbursts and difficult relations to his peers in school. Speech delay at age 18 months, was initially ascribed to repeated ear infections, but sensorineural hearing impairment was subsequently diagnosed and treated with hearing aids as he did not fulfill criteria for having CI until age 6 when he had left-sided CI. Around the same time, he had learned sign language, which he tends to prefer as his communication modus. There is no suggestion of neurological, dystonic nor visual problems. Current schooling is in college learning tradesman skills.

**Figure 2:**
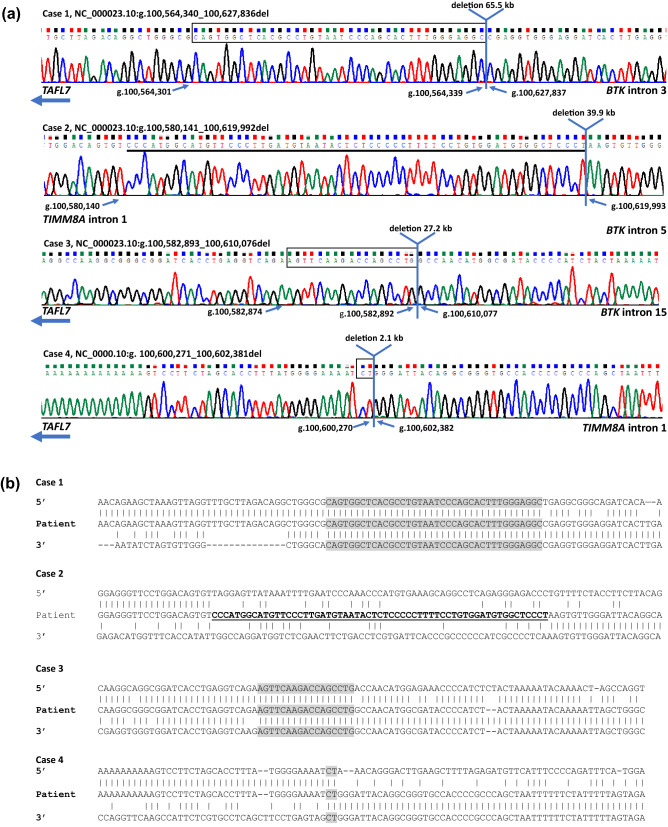
Breakpoint junctions and flanking regions identified in case 1–4. (**a**) Sequence chromatograms are shown with the identified breakpoint spanning region above. Regions of microhomology are shown in black boxes for case 1, 3 and 4. However, it cannot be determined which end of the overlapping region is the real breakpoint. The 59 bp inverted insertion identified in the breakpoint region in case 2 is underlined. (**b**) Sequences of the identified breakpoint junctions in case 1–4 compared to upstream and downstream references sequence. Regions of microhomology are shown in grey.

Through array-CGH a 17–19 kb de novo deletion with breakpoints in intron 1 of *TIMM8A* and intron 5 of *BTK* was detected. However, breakpoint spanning PCR, based on the array-CGH result, was not successful. Accordingly, through mate-pair sequencing and breakpoint spanning PCR, a larger rearrangement was detected, involving a 39.9 kb deletion (ChrX: NC_000023.10:g.100580141_100619992del) including the entire coding region of *TIMM8A* and exon 6–19 of *BTK*. Furthermore, a 59 bp inverted insertion was observed in the breakpoint region (Fig. 2). The insertion corresponded to a region, mapping to chrX:100, 582, 138–100, 582, 196, which was located within the deleted sequence. The proximal breakpoint of the deletion was located in a LINE (long interspersed element) and the distal breakpoint was located in a SINE. Both breakpoints of the 59 bp insertion were located within a LINE.

#### *Case 3* (XLA-MTS)

Italian male patient, previously published in 2004^16^, presenting clinically with severe hearing impairment from 2 years of age, marked reduction in visual acuity, XLA, and later in life dystonia became present. Subsequent follow-up investigations revealed a skewed X-inactivation pattern in the mother, who also had clinical symptoms including dystonia. Since the original report the patient and his mother are both deceased. Pizzuti et al., by means of successive PCR, demonstrated that the deletion included both exons of *TIMM8A* and a partial deletion of *BTK* (the telomeric breakpoint was located between *BTK* exon 7 and 19)^16^. We carried out further successive PCR testing, which demonstrated that *BTK* exon 15 and the first 788 bp of intron 16 are intact, while exon 16 could not be amplified. Finally, through mate-pair sequencing and breakpoint spanning PCR, a 27.2 kb deletion (chrX: NC_000023.10:g.100582893_100610076del) including the entire coding region of *TIMM8A* and exon 16–19 of BTK was identified. The breakpoints were both located in SINEs. The deletion was maternally inherited, as evidenced through PCR and breakpoint sequencing.

#### *Case 4* (MTS)

Dutch male patient, born in 1986, briefly mentioned in Tranebjærg, 2013, as patient 35 in Table 1^5^. Clinically the boy presented with congenital deafness, abnormal VEP (visual evoked potential) response and reduced visual acuity, as well as progressive ataxia. No dystonia was observed. Through successive PCRs a 2.1 kb deletion (chrX: NC_000023.10:g.100600271_100602381del) spanning exon 2 of *TIMM8A* was identified. The telomeric part of the deletion was located in a SINE. As evidenced through PCR and breakpoint sequencing, the deletion was maternally inherited, and the affected brother of the mother also carried the deletion. The grandmother, having an affected son and a carrier daughter, should be an obligate carrier; however, the deletion could not be detected by PCR of DNA purified from blood, suggesting germline mosaicism.

### Verification of identified breakpoints

For each of the four cases the breakpoints were validated at base pair level resolution, and all junctions were confirmed by Sanger sequencing. In Case 1, 3 and 4 junctional microhomology was observed, ranging in size from 2 to 38 nucleotides. In Case 2 a 59 bp inverted insertion was observed in the junctional region, corresponding to position chrX:100, 582, 138–100, 582, 196 (Fig. 2).

## Discussion

In this study, we describe four patients of which one had MTS due to a 2.1 kb deletion involving exon 2 of *TIMM8A*, and three had XLA-MTS due to larger contiguous gene deletions involving *BTK* as well as *TIMM8A* (Table 1). Patients typically present with progressive hearing impairment in their first years of life, and additional neurological symptoms including dystonia and optic atrophy developing later. From a clinical standpoint, early detection of patients with pathogenic variants in *TIMM8A* is important to guide parents and optimize patient treatment. It must be expected that new cases with congenital or early onset hearing impairment of the postsynaptic auditory neuropathy type, due to *TIMM8A* pathogenic variants, may be genetically diagnosed before they present with visual and neurological symptoms^9^.

**Table 1:** Clinical and molecular features of the four MTS cases identified.

Subject	Case 1	Case 2	Case 3	Case 4
Nationality	Australian	Scottish	Italian	Dutch
Sex and age (2021)	Male (16 y)	Male (21 y)	Male (21 y)	Male (35 y)
Phenotype	XLA-MTS^a^	XLA-MTS^b^	XLA-MTS^c^	MTS^d^
Hearing impairment	Profound hearing impairment	Deafness	Severe hearing impairment	Congenital deafness
Cochlear implant	Yes	Yes (at age 6 y)	NI	NI
Other	Behavioral abnormalities	Some behavioral abnormalities	Dystonia	Progressive ataxia
Visual acuity	N (at age 6)	N (at age 9 y)	Marked reduction in visual acuity	Reduced visual acuity
Size of deletion	~ 63.5 kb	~ 39.9 kb	~ 27.2 kb	~ 2.1 kb
Location on chrX:	g.100564340_100627836del	g.100580140_100619992del	g.100582893_100610076del	g.100600271_100602381del
Heritance of deletion	Probably de novo (mother of the proband did not harbor the deletion)	De novo	Maternally inherited	Maternally inherited (affected brother also carried the deletion)
ClinVar ID	SCV001999951	SCV001999952	SCV001999953	SCV001999954

^a^Briefly mentioned in Tranebjærg, 2013, as patient 33; ^b^Briefly mentioned in Tranebjærg, 2013, as patient 34; ^c^Clinical details published in Pizzuti et al. 2004, ^d^Briefly mentioned in Tranebjærg, 2013, as patient 35. NI = no information; N = normal; y = years.

Molecular mechanisms responsible for non-recurrent deletion events, as seen in MTS and XLA-MTS, can be explained by non-homologous end joining (NHEJ) or homologous recombination, which are catalyzed by the presence of repeat elements (eg. LINEs and SINEs)^17,18^. In two of the cases the deletions were large (63.5 and 27.2 kb in case 1 and case 3, respectively), breakpoints were located in SINEs, and correspondingly the junctional regions were characterized by long stretches of microhomology, indicating that these events have occurred by homologous recombination. On the other hand, one of the deletions (case 4) exhibited a much smaller 2 bp region of microhomology. Apart from this overlap, the region was not characterized by extensive stretches of homology. A similar pattern is seen in two of the three events described by Arai et al., where the deletion breakpoints also reside outside of repeat elements, although deletion size was considerably larger (149.7 and 196 kb), compared to the 2.1 kb deletion observed by us^11^. This type of rearrangement could arise through FoSTeS due to a replication based mechanism^17^. In this study, SINE and LINE elements and microhomology seem to be involved in the identified gross deletions. However, other mechanisms may also be involved as evidenced by case 2, where the inverted insertion of a 59 bp fragment, originating from within the deleted region, was observed. The event is more complex with the involvement of at least four breakpoints, resembling a mild form of germline chromothripsis, where the size of the shattered fragments can vary from few bases to several megabases^19^. The underlying mechanisms explaining this event could be homologous recombination with the presence of inverted repeats^18^ or microhomology mediated mechanisms.

In conclusion, our study adds one MTS and three XLA-MTS deletion patients to the list of at least 69 published cases^9^. Using NGS technology Wang et al. identified three patients with early auditory neuropathy due to variants in *TIMM8A*, and in one XLA-MTS was diagnosed upon detection of a 16.334 bp deletion. In total nine out of the 69 published cases had a deletion involving *TIMM8A*. Although there might be a bias, based on different molecular genetic methods used, it appears that deletions involving *TIMM8A* are less frequent compared to smaller loss-of-function variations, most likely because larger deletions are more deleterious and are negatively selected during embryonal development. Still, it is important to carry out copy number detection in patients where MTS is suspected. Mate-pair library generation combined with NGS analysis is a fast and efficient method for identification of deletion breakpoints located in genomic regions with a high density of repetitive elements. The longer insert size in mate-pair sequencing makes mapping across repetitive regions feasible, and the discrimination of concordant versus dis-concordant reads enables mapping of both balanced and unbalanced events. In all four cases, mate-pair sequencing enabled amplification and Sanger sequencing of the deletion breakpoints, which makes this approach attractive in patients with suspected MTS or MTS-XLA.

## Material and methods

### Ethical statement

All participants, or their legal guardian, provided written informed consent for participation in the study. The study was approved by Regional Research Ethical Committee, Copenhagen (KF 01–234/02). All methods were carried out in accordance with relevant guidelines and regulations.

### Successive PCR testing for identification of genomic breakpoints

Successive PCR testing was carried out to identify the presence of a deletion and to narrow down the location of deletion breakpoints. First, PCR amplification of the two exons and the intronic region of *TIMM8A* (NM_004085.4), then, depending of the result, amplification of exon 5, 6, 7, 10, 13, 15, 16, and 17 of *BTK* (NM_000061.3), and first exon of *TAF7L* (NM_024885.4) was carried out. Subsequently, eleven additional primer set were tested: two of them positioned in *BTK* intron 15 and nine of them in sequence between the *TIMM8A* coding region and *TAF7L* exon 1. Primer sequences and PCR conditions are available in Supplemental Table 1. PCR products were sequenced using BigDye Terminator chemistry (Applied Biosystems) and sequenced on an ABI 3130XL genetic analyzer (Applied Biosystems).

### Next-generation mate-pair sequencing

Mate-pair libraries of DNA from case 1, 2 and 3 were prepared using the Mate Pair Library v2 kit (Illumina, San Diego, CA, USA) essentially according to the manufacturer’s protocol. Libraries were quantified and sequenced on an Illumina Genome Analyzer, GAII. Reads were aligned to the human genome using Burrows Wheeler Aligner (BWA)^20^. Discordant reads were extracted and structural variations were identified using SVDetect^21^. All genomic coordinates in the study were based on the GRCh37/hg19 assembly.

### Verification of identified breakpoints

Mate pair identified breakpoints were assessed by PCR and subsequent Sanger sequencing (Fig. 2). PCR was carried out on genomic DNA from the proband and from a normal control (Case 1: 2008F: 5'-AAGTGCTGCTTCTGCCAGAACAAAAGACC-3' and 3981R: 5'-CCAGAAAGACAGATTCCGGTAAGAAGAGACC-3'; Case 2: 2768F: 5’-ACAGTGTTTAGAACAATCCTGGCACATAGAAA-3’ and 4310R: 5’-TAGCCACAGACTCTCTCT TTCCATTTCCTTAA-3’; Case 3: 18344F: 5'-AGAAGAAGAAGGTGGATCATGGCAGACG-3' and 22568R: 5'- GAATCAGAGCAGTCACTCACTCCCAACC-3' and 6377R: 5'-CCGCCTCCTTTCCTC TAGGCATGTA-3' (nested); Case 4: int1-F1: 5´-TAGGAGGGCGTGTGGTTAAG-3´ and Del2-R2: 5´-GGAAGCTTAGTTCATCCAGGTC-3´). PCRs were performed using AccuPrime Taq DNA polymerase, high fidelity (ThermoFisher Scientific). PCR conditions are available upon request. To visualize microhomology and/or indels, the junction sequences flanking the breakpoints were aligned to genomic sequence using MultAlin (http://multalin.toulouse.inra.fr/multalin/; Fig. 2).

## Supplementary Information

Supplementary Information.
