# Supplementary material for: Identification and analysis of deletion breakpoints in four Mohr-Tranebjærg syndrome (MTS) patients
Source: Sci Rep. 2022 Sep 2;12:14959. doi: 10.1038/s41598-022-18040-y (PMC9440042; doi:10.1038/s41598-022-18040-y)
Supplement: Supplementary file 1 — Supplementary Information. [file 41598_2022_18040_MOESM1_ESM.docx]

# Identification and analysis of deletion breakpoints in four

# Mohr-Tranebjærg syndrome (MTS) patients

^1^Nanna Dahl Rendtorff, ^1^Helena Gásdal Karstensen, ^1^Marianne Lodahl, ^2^John Tolmie†, ^3^Catherine McWilliam, ^4,5^Mads Bak, ^4,5^Niels Tommerup, ^4,5,6^Lusine Nazaryan-Petersen, ^7,8^Dirk Kunst, ^9^Melanie Wong, ^10^Shelagh Joss, ^11,12^ Valerio Carelli, and ^1,13^Lisbeth Tranebjærg

1. Department of Clinical Genetics, Center of Diagnostics, Copenhagen University Hospital - Rigshospitalet, Copenhagen, Denmark
2. Clinical Genetics Service, Laboratory Medicine Building, Southern General Hospital, Glasgow, Scotland
3. Clinical Genetics, Human Genetics Unit, Ninewells Hospital, Dundee, Scotland
4. Wilhelm Johannsen Center for Functional Genome Research, University of Copenhagen, Copenhagen, Denmark
5. Department of Cellular and Molecular Medicine, University of Copenhagen, Copenhagen, Denmark
6. Center for Genomic Medicine, Copenhagen University Hospital, Rigshospitalet, Copenhagen, Denmark
7. Department of Otorhinolaryngology, Head and Neck Surgery, Radboud University Nijmegen Medical Centre, Nijmegen, The Netherlands
8. Donders Institute for Brain, Cognition and Behaviour, Radboud University Nijmegen, Nijmegen, The Netherlands
9. Department of Allergy and Immunology, The Children’s Hospital at Westmead, Sydney, Australia
10. West of Scotland Centre for Genomic Medicine, Queen Elizabeth University Hospital, Glasgow, United Kingdom
11. IRCCS Istituto delle Scienze Neurologiche di Bologna, Programma di Neurogenetica, Bologna, Italy.
12. Unit of Neurology, Department of Biomedical and NeuroMotor Sciences (DIBINEM), University of Bologna, Bologna, Italy.
13. Institute of Clinical Medicine, University of Copenhagen, Copenhagen, Denmark

† John Tolmie has passed away.

**Running Title**: Deletion breakpoints in four Mohr-Tranebjærg syndrome patients

***Correspondence**: Lisbeth Tranebjærg (E-mail: tranebjaerg@sund.ku.dk), Nanna D. Rendtorff (E-mail: nanna.dahl.rendtorff@regionh.dk)

# Supplementary Tabel 1 Primer sequences and PCR conditions

| Primer name | Position^a^ | Primer sequence (5’→3’) | Product length (bp) | | PCR^b^ annealing temp (°) |
| --- | --- | --- | --- | --- | --- |
| Ex5F BTK  Ex5R BTK | 100,625,195-100,625,215  100,624,902-100,624,921 | AACCTGAACACCATTGCTGAC  CTTCTTTCCTTTTCCTCCCG | 314 | 59-61 | |
| Ex6-7F BTK  Ex6-7R BTK | 100,617,728-100,617,747  100,617,065-100,617,084 | AGAGGAAAACATGCAAATGG  CCAAGTCCCAGGGTAATTCT | 683 | 59-61 | |
| Ex10F BTK  Ex10R BTK | 100,614,522-100,614,541 100,614,212-100,614,230 | TGCCTGCTACTCTCCATTTC  AACAGGCCCTCAGTTCAAG | 330 | 59-61 | |
| Ex13F BTK  Ex13R BTK | 100,612,715-100,612,734  100,612,366-100,612,385 | TGAGAGGAGAAACCTCTGGA  ATCTGTCTTGAGCGTCCTTG | 369 | 59-61 | |
| Ex15F BTK  Ex15R BTK | 100,611,353-100,611,372 100,610,870-100,610,889 | CTGGTGTGACCCCTTATCTG  TATTTGATGGGCTCAGCACT | 503 | 59-61 | |
| BTK int 15F_1  BTK int 15R_1 | 100,610,747-100,610,766 100,610,468-100,610,487 | CCATTCTGGATTTGGAACAG  GGAAATGGAGAGTACATGCC | 299 | 56-61 | |
| BTK int15F_3  BTK int15R_3 | 100,610,465-100,610,485 100,610,251-100,610,271 | CATGTACTCTCCATTTCCTGC  CGAGACTCCATCTCAAAACAA | 235 | 56-61 | |
| BTK ex 16F  BTK ex 16R | 100,609,842-100,609,861 100,609,492-100,609,511 | CGTGGTGGAAATGAATCAAA  AGATCGGCAGAAAACGCTAG | 370 | 59-61 | |
| BTK ex 17F  BTK ex 17R | 100,609,066-100,609,085 100,608,728-100,608,747 | TTTGAGAAAGCCTGTGTGTG  GTAAGCACTCCCCAAGGATT | 358 | 59-61 | |
| TIMM8A-ex1-F | 100,603,755-100,603,776 | CTCGCGCCGACGCAGTGCACTC | 346 | 60 | |
| TIMM8A-ex1-R | 100,603,431-100,603,452 | CCCGAATCCCCGACGTTGTCGC |  |  | |
| TIMM8A-var2-F | 100,603,714-100,603,733 | GCTAGCTGTGGTTCCGGTTC | 507 | 60 | |
| TIMM8A-var2-R | 100,603,227-100,603,246 | AGCACTAGGCAGCTTCAACC |  |  | |
| TIMM8A int1 F1 | 100,602,567-100,602,586 | TAGGAGGGCGTGTGGTTAAG | 683 | 62 | |
| TIMM8A int1 R1  TIMM8A int1 F2 | 100,601,924-100,601,943  100,603,575-100,603,596 | ATCATAGGGCCAGAAAATGG  GCAGCATTTCATCGAGGTAGAG | 688 | 62 | |
| TIMM8A int1 R2  TIMM8A int1 F3 | 100,602,909-100,602,929  100,603,105-100,603,126 | AAATACCTTCCACCCTTTTGC  CCTCGGGATTTACCTTCATTAC | 588 | 62 | |
| TIMM8A int1 R3 | 100,602,539-100,602,559 | TCACACATGTCAGGAAGAAGATG |  |  | |
| TIMM8A int1 F4 | 100,602,639-100,602,660 | TTAACTCTGGTCTGCCTTGGAC | 652 | 62 | |
| TIMM8A int1 R4 | 100,602,009-100,602,030 | TGGTTTTAACTCCACAATGCTG |  |  | |
| TIMM8A-ex2-F | 100,601,791-100,601,813 | TTGCTATATACTTGGTCAGCTGC | 436 | 60 | |
| TIMM8A-ex2-R | 100,601,378-100,601,399 | AGTAACAAAAGATGGGAGCCAA |  |  | |
| TIMM8A 3-UTR-F3 | 100,601,577-100,601,597 | TGTTTTGTGAACTGCGTTGAG | 623 | 62 | |
| TIMM8A-3 UTR-R3 | 100,600,975-100,600,999 | AAGGGAGATCAAGATAACTGAAGTG |  |  | |
| TIMM8A 3-UTR-F4 | 100,601,165-100,601,189 | TTCCTATAATTTTGATAGTGGGACC | 598 | 62 | |
| TIMM8A-3 UTR-R4 | 100,600,592-100,600,611 | TAAATCTCTCCGGGTTGCAG |  |  | |
| Frag1F TIMM8A  Frag1R TIMM8A | 100,600,574-100,600,593 100,600,444-100,600,463 | TAGCATGGGCTGATTCCAAG  AGCACATAACCGCTTCCCTC | 150 | 59-61 | |
| Frag 3 F | 100,600,244-100,600,265 | TTCCCCATAAAGGTGCTAGAAG | 385 | 60 | |
| Frag 3 R | 100,599,881-100,599,904 | CGGCACAGAGTATGAACTTAAATG |  |  | |
| Frag 4 F | 100,597,681-100,597,702 | GTGTTTTGACACTACTGGGCAC | 316 | 60 | |
| Frag 4 R | 100,597,387-100,597,408 | CACCAGAGTAGCTTGGCTTTTC |  |  | |
| Frag 5 F | 100,591,602-100,591,621 | AGATGGCCCTATTGCAGTCC | 496 | 60 | |
| Frag 5 R | 100,591,126-100,591,148 | TCACACATGTCAGGAAGAAGATG |  |  | |
| 12980F TIMM8A  13229R TIMM8A | 100,590,689-100,590,708 100,590,440-100,590,459 | GATTCTCATACCCATCCAGA  TGCAAGGTAGACATTGAAGA | 269 | 59-61 | |
| 13641F TIMM8A  13863R TIMM8A | 100,590,027-100,590,047 100,589,805-100,589,825 | TAAATGTACAGCTGAACTTGC  TCATGTAACCAAATACCACCT | 243 | 59-61 | |
| DDP3-U | 100,580,280-100,580,300 | GTTGGGAGTTTCAAAGGACAA | 289 | 60 | |
| DDP-L | 100,580,012-100,580,032 | TTGTGAGGGATATGTCTCTCT |  |  | |
| TAF7L ex1F  TAF7L ex1R | 100,548,000-100,548,019 100,547,586-100,547,605 | GGGACAGCTCCCCATTTCTT  TAACACGATCGGTTGCTCCC | 434 | 59-61 | |

^a^ The physical position according to Genome Browser Assembly Feb 2009 (GRch37/hg19). ^b^ PCR conditions for PCR-reactions with Ampliqon taq DNA polymerase were: 5 min at 94°C, followed by 35 cycles of 30 sec at 94°C, 30 sec at the individual annealing temperature for each primer pair, 30 sec at 72°C, and a final extension for 10 min at 72°C.
